# Subjective face recognition difficulties, aberrant sensibility, sleeping disturbances and aberrant eating habits in families with Asperger syndrome

**DOI:** 10.1186/1471-244X-5-20

**Published:** 2005-04-12

**Authors:** Taina Nieminen-von Wendt, Juulia E Paavonen, Tero Ylisaukko-Oja, Susan Sarenius, Tiia Källman, Irma Järvelä, Lennart von Wendt

**Affiliations:** 1 Department of Child Neurology, Hospital for Children and Adolescents, Helsinki University Central Hospital, Helsinki, Finland; 2 Department of Child Psychiatry, Hospital for Children and Adolescents, Helsinki University Central Hospital, Helsinki, Finland; 3 Department of Molecular Medicine, National Public Health Institute, Helsinki, Finland; 4 Department of Medical Genetics, University of Helsinki, Helsinki, Finland; 5 Laboratory of Molecular Genetics, Helsinki University Hospital (Laboratory Services), Helsinki, Finland

## Abstract

**Background:**

The present study was undertaken in order to determine whether a set of clinical features, which are not included in the DSM-IV or ICD-10 for Asperger Syndrome (AS), are associated with AS in particular or whether they are merely a familial trait that is not related to the diagnosis.

**Methods:**

Ten large families, a total of 138 persons, of whom 58 individuals fulfilled the diagnostic criteria for AS and another 56 did not to fulfill these criteria, were studied using a structured interview focusing on the possible presence of face recognition difficulties, aberrant sensibility and eating habits and sleeping disturbances.

**Results:**

The prevalence for face recognition difficulties was 46.6% in individuals with AS compared with 10.7% in the control group. The corresponding figures for subjectively reported presence of aberrant sensibilities were 91.4% and 46.6%, for sleeping disturbances 48.3% and 23.2% and for aberrant eating habits 60.3% and 14.3%, respectively.

**Conclusion:**

An aberrant processing of sensory information appears to be a common feature in AS. The impact of these and other clinical features that are not incorporated in the ICD-10 and DSM-IV on our understanding of AS may hitherto have been underestimated. These associated clinical traits may well be reflected by the behavioural characteristics of these individuals.

## Background

Asperger Syndrome (AS), which belongs to the autism spectrum disorders, is a behavioural disorder defined by problems in social interaction, circumscribed unusual interests, repetitive routines and rituals, speech peculiarities and non-verbal communication problems usually without language delay. The overall cognitive level is generally within normal limits and motor clumsiness may also be seen [1-5].

The diagnosis of AS is a clinical one, based on a set of symptoms and signs, emphasizing development and behaviour in childhood, despite the fact that the diagnosis can also be made in adulthood. The definite diagnosis is based on the ICD-10 [3] and DSM-IV [4] (APA, 1994) classifications, which comprise the current official diagnostic criteria for AS.

In addition to the official diagnostic criteria for autism spectrum disorders listed in the ICD-10 [3], and DSM-IV [4], there appear to be some additional clinical features, which may be over-represented in these disorders. Difficulties of face recognition has been reported in a number of individuals with AS [6-9]. It has been suggested that face recognition problems may play a role as an essential feature in AS [6,9] and therefore could play a causal role for social dysfunction [6]. Njiokiktjien et al. [7] described the co-existence of face recognition difficulties and disordered recognition of emotions in individuals with AS thereby suggesting an impact of this feature on the social shortcomings characteristic for this disorder.

Barton et. al. [10] showed that there is heterogeneity in social developmental disorders (SDD), like AS and SEPD (social-emotional processing disorder) what it comes to face perception and recognition. They showed that a subgroup of persons with SDD performed normally on face perception and recognition, while those who did not could be divided into two subgroups. The other subgroup had poor perception of facial structure but relatively preserved imagery, while the other subgroup had better perception than imagery. The authors judged that the social disturbance in SDD does not invariably lead to impaired face recognition; meaning that the heterogeneity makes it less likely that poor social function was the cause of impaired face recognition [10].

The official diagnostic criteria (ICD-10, DSM-IV) [3,4] do not include sensory difficulties as a criterion for AS. However, already Hans Asperger [11] recognized sensory aberrations in children with AS. The children he described displayed a range of hyposensitivities and hypersensitivities to taste, tactile and auditory stimuli [11]. More recently, consideration has been given to the possibility that sensory processing is an underlying feature of AS, also reflected in their ability to conduct daily life successfully. Some evidence even suggest that difficulties in socialization can be linked to poor sensory processing as e.g Myles et al. [12] in their study concluded that temper tantrums in AS is due to an overload of sensory stimuli.

The sensory processing issue in AS has more extensively been addressed by Dunn et al. [13,14] using The Sensory Profile test which consists of a 125-item questionnaire, that describes responses to sensory events in daily life. The children with AS studied by them were normal on modulation on visual input affecting emotional responses. This implies that the children with AS can process visual-perceptual information in context, and perhaps can orient to social situations using their visual system in a similar way to other children, even though the other behavioural responses might be different [12-14]. Furthermore, it was shown that persons with AS were hyperresponsive, (detecting texture, taste and temperature differences), and that they had difficulty quieting down for sleep because they would have high detection of stimuli to grab their attention [13]. On the basis of these studies it seems that children with AS have difficulties in sensory modulation; and that the stereotypic behaviours serve as an organizing function [13]. Dunn et al. [13,14] suggested that sensory issues are important in AS and that including sensory processing status in diagnostic criteria might be warranted.

Concerning auditory processing Myles et al. [12] and Dunn et al. [13,14], showed that poor auditory processing in children with AS is associated with attention challenges. This was interpreted by the authors as showing children with AS having difficulties in capturing the auditory information in social conversations. The results of the study by Jansson-Verkasalo and co-workers [15] is in line with this as they demonstrated that children with AS exhibited difficulties in discriminating tone pitch. The findings suggest according to Jansson-Verkasalo et al. [15] that auditory sensory processing is deficient in children with AS.

Abnormal sleep patterns have been reported in up to 65 percent of children with autism spectrum disorders [16]. According to the study by Paavonen et al. [17] behavioural, attention and social problems in children with AS are aggravated when a sleep disorder is present. Interestingly, an open clinical trial on 15 children with AS aged 6–17 years showed that melatonin improved the sleep patterns in all individuals with AS, and half of them displayed excellent responses [17]. In a hospital-based series by Tani et al. [18] on twenty adults with AS, the sleep questionnaire revealed insomnia in 90 percent and the sleep diary in 75 percent. According to Tani et al. [18] the neuropsychiatric deficits inherent in AS predispose both to insomnia, anxiety and mood disorders. These two studies suggest that sleep difficulties inherent in AS are preserved from childhood to adulthood. This data might be useful diagnostically, and hence determining their prevalence might be of interest, even if they are not related to sensory processing.

According to Gillberg and Råstam [19] and Gillberg and Billstedt [20], special eating habits, such as particular food refusal, food fads, pica, hoarding, overeating and various degrees of anorectic behaviour, including complete food refusal and compulsive ordering of food on the plate, are involved in autism. In line with this are the results of the study by Sobanski et al. [21] who in a hospital-based series of children with AS (age-span 7–l9 years), demonstrated reduced weight gain in all males and anorectic-like eating behaviour in four out of 36. A similar result was reached by Bölte et. al. [22] in a hospital-based series of 103 individuals (74 males and 29 females; age-range 10.1–39.9 years), of whom 71 had been diagnosed with autism and 32 with AS. It was shown that 28 percent of the males with AS had a body mass index (BMI) in the fifth percentile or below. However, none fulfilled the ICD-10 criteria for anorexia nervosa. The only factor that significantly influenced BMI was hyperactivity and it was concluded that the co-occurrence of autism spectrum disorders and anorexia nervosa was due to chance [22].

As there is direct and indirect evidence that a number of clinical features related to sensibility, not used as official criteria in the DSM-IV [4] or ICD-10 [3] for AS occur fairly frequently in this syndrome, the present study was undertaken in order to determine whether these are associated with AS in particular or whether they are merely a familial trait that is not related to the diagnosis.

## Methods

### Subjects

The present study is part of larger study of autism spectrum disorders. The total clinical series comprises 250 families (1360 individuals). The Asperger family series in this large series was recruited through the Hospital for Children and Adolescents, Department of Child Neurology, Helsinki University Central Hospital (HUCH), and the Helsinki Asperger Center (HAC), Medical Center Dextra, Helsinki, Finland. The Department of Child Neurology at the Helsinki University Central Hospital primarily serves the catchment area of the Helsinki University Central Hospital (population 1.4 million), but it also serves the entire country as a tertiary referral unit. The HAC is a private clinic, freely available to anyone and serving the whole of Finland (population 5.2 million).

The Asperger clinical series consisted of 29 families, in which AS was present in at least two generations. From these families, 10 large families, in which the diagnosis was present in at least two generations exclusively on either the maternal or the paternal side and for which molecular genetic data had also been obtained, were recruited for the present analysis after informed consent had been obtained [26]. Large kindred were chosen, as including only small families would have compromised the statistical analyses. These ten families consisted of 138 persons, of whom 58 individuals were found to have a confirmed diagnosis of AS using the diagnostic procedure outlined above (range 4.5–78.2 years; mean 32.8 years; SEM 2.8; CI 27.1–38.5). Another 56 (range 3.5–77.9 years; mean 33.8 years; SEM 2.6; CI 28.2–38.5) also went through the diagnostic procedure and were found not to fulfill the criteria for AS. The remaining 24 family members could not be included in the study, as four lived abroad and the others did not agree to participate. This means that 83% of the family members participated.

### Protocol

The individuals investigated in this study were diagnosed as representing AS if they fulfilled both the diagnostic criteria of the ICD-10 [3] and the DSM-IV [4]. For this purpose the following instruments were used:

The Asperger Syndrome Diagnostic Interview (ASDI) [23] is an investigator-based interview consisting of 20 items, all of which must be covered in detail. The interviews were conducted in accordance with the principles of this instrument.

The Autism Diagnostic Observation Schedule (ADOS) is a semi-structured, standardized assessment of communication, social interaction and play or imaginative use of materials for individuals who have been referred because of possible autism spectrum disorders [24].

The Autism Diagnostic Interview – Revised (ADI-R) [25] is an investigator-based interview containing structured coding for each behavioural item. It consists of six sections dealing with background orientation, developmental history and earlier and current behaviour.

#### Assessment of clinical features related to face processing, sensibility, sleeping and eating not used as official criteria in the DSM-IV or ICD-10

For this study, a particular set of questions the Asperger syndrome related additional features questionnaire (ASFQ) (Appendix 1) was developed in order to determine whether there were any defined data showing abnormalities in the domains of face recognition difficulties, presence of aberrant sensibilities, sleeping disturbances, and aberrant eating habits. The questions were presented during an interview and the answers were recorded by the interviewer as yes or no. The interviewers were two neuropediatricians and two nurses trained in autism spectrum disorders, all of them had a long experience in autism spectrum disorders. In order to increase the validity pre-constructed clarifying examples and additional instructions for each of the questions were used.

In addition to this available medical charts and other relevant documentation were scrutinized for previously set diagnoses, special examinations, and developmental history.

### Statistical methods

First, researches conducted a frequency analysis, and secondly Mantel-Haentzel pooled risk estimates were calculated. Finally, logistic regression models using the generalized estimating equations procedure were developed (random effects model linear model). This method is similar to ordinary logistic regression, but it takes account of the possible dependences between the family members.

## Results

The prevalence of face recognition difficulties emerged as being over-represented in the family members with AS in this study, as the percentage of occurrence was 46.6 percent compared with 10.7 percent in the family members without AS (Table 1). The prevalence of self-reported aberrant presence of sensibilities was significantly higher (OR, Mantel-Haentzel 38.27 (95% CI 6.29–232.77) in the individuals with AS than in their non-AS family members (Table 2). Among the subcomponents of aberrant sensibility; touch, light, sounds and smell emerged as being significantly altered in the group of family members with AS (Table 1). Other features which were significantly more frequent in family members who fulfilled the criteria for AS were sleeping disturbances and aberrant eating habits (Tables 1, 2).

**Table 1:** Mantel-Haentzel (MH) pooled risk estimates (risk of the symptom in Asperger individuals)

	Overall prevalence % (n)	Prevalence in controls % (n)	Prevalence in cases % (n)	OR_MH _(95% CI)
Face recognition difficulties	28.9 (33)	10.7 (6)	46.6 (27)	20.46 (4.51–92.88)
Presence of altered sensibilities	69.3 (79)	46.4 (26)	91.4 (53)	38.27 (6.29–232.77)
- of touch	31.6 (36)	8.9 (5)	53.4 (31)	12.16 (3.37–43.97)
- of light	25.4 (29)	7.1 (4)	43.1 (25)	7.57 (2.13–26.87)
- of sounds	33.3 (38)	16.1 (9)	50.0 (29)	5.59 (2.08–15.01)
- of smell	34.2 (39)	23.2 (13)	44.8 (26)	2.56 (1.01–6.47)
- of pain	11.4 (13)	7.1 (4)	15.5 (9)	2.54 (0.65–9.96)
Sleeping disturbances	36.0 (41)	23.2 (13)	48.3 (28)	3.11 (1.24–7.78)
Aberrant eating habits	37.7 (43)	14.3 (8)	60.3 (35)	8.41 (2.80–25.27)

OR = Odds Ratio

**Table 2:** Logistic regression models (risk of the symptom in Asperger individuals)

	Model coefficient/ parameter estimate	SE	p-value
Face recognition difficulties	2,26	0,56	<0,001
Presence of altered sensibilities	2,70	0,58	<0,001
- of touch	2,42	0,54	<0,001
- of light	2,03	0,56	<0,001
- of sounds	1,61	0,44	<0,001
- of smell	1,00	0,42	0,017
Sleeping disturbances	1,11	0,41	0,007
Aberrant eating habits	2,24	0,48	<0,007

The results of the random effect models are presented in Table 3. They further confirm that face recognition difficulties (p < 0.001), aberrant sensibilities (p < 0.001), and aberrant eating habits (p = 0.007) are highly typical of AS. The risk of sleep problems (p < 0.007) is also slightly higher in persons with AS.

The sensitivity for the presence of aberrant sensibility in the family members with AS was 0.91, whereas the specificity was 0.54. The corresponding figures for face processing difficulties were 0.47 and 0.89, for sleeping disturbances 0.48 and 0.77 and for aberrant eating habits 0.60 and 0.86. The aberrant sensibility items had a higher sensitivity, they also had lower specificity, and that actually all the items had a very similar data value. Hence the differences between the items are due to a criterion shift, with all subjects having a greater bias to answer yes to the aberrant sensibility items.

## Discussion

The diagnostic criteria for autistic disorders used in the ICD-10 and DSM-IV manuals apparently represent the experts' generally accepted views of the clinical delineation of AS at the time the latest revision was made. One evident drawback of these sets of diagnostic criteria is that the distribution of symptoms and signs underlying the criteria in an average population has not been satisfactorily outlined. The same also applies to many of the sets of screening questionnaires and other instruments used in the diagnostic process. It can even be questioned whether currently used instruments actually cover the core features of AS. Using several such instruments and including only individuals who fulfilled both ICD-10 and DSM-I0 criteria for AS, as was the case in this study should improve the detection rate of the features typical for AS.

The data was collected by a few experienced investigators using a standardized procedure that is expected to increase the reliability of the results. The same sets of diagnostic instruments were used for each age group to facilitate the comparison of the results. The chosen set of diagnostic instruments was considered to have several advantages including ADI-R [25] that covers the entire lifetime. Using different set of diagnostic instruments for each age group would have hampered the comparability of the results. The only new method used in this study was the Asperger syndrome related additional features questionnaire (ASFQ)(Appendix 1), which was designed specifically for the purpose of detecting additional clinical feature much in the same way as in ICD-10 and DSM-IV. This instrument was not validated, but in a pilot-like setting to find out whether family members with or without AS differed from each other it was considered as a suitable method. Another drawback of the ASFQ method is that the results are based on self-reported perceptions that are not objective. The relevance of ASFQ needs to be studied in a control group of average population before its applicability as a diagnostic instrument for AS can be assessed.

The families chosen for this analysis may not necessarily completely reflect the average AS population as we for analytical reasons focused on large pedigrees with several AS individuals. Nor is the series unequivocally a population-based one, as the families were recruited from originally clinical series in which the index case had been referred for a diagnostic evaluation. By using non-AS individuals from the same families as a comparison group, we think that the effects of the family social environment are eliminated at least to some extent. The methodological weakness need to be taken into account, but the results seem to be fairly straightforward, as face recognition difficulties and aberrant sensibility emerge as being fairly typical of AS.

The basic aim of the present research project on AS was to elucidate the pathogenesis, especially with respect to the molecular genetic issues [26]. As AS is a complex disorder it seems likely that the discovery of endophenotypes could be of help for the identification of both putative susceptibility genes and specific neuroimaging features. In line with this, it seems attractive to speculate that our findings indicate the existence of a general sensory processing abnormality in AS as these individuals frequently are sensitive to noise, bright lights, strong smells and touch. Thus, these findings may be useful as well for diagnostic purpose as for understanding of the pathogenesis. Our findings are supported by Dunn et al. [13,14] who considered that the contributions of sensory processing status in diagnostic criteria might be warranted. Another possibility is that individuals with AS can or should be sub-grouped on the basis of their pattern of sensory processing status, sleeping and eating problems.

In the case of face recognition difficulties, there are no data on its occurrence in the average population, but it is not unlikely that the non-AS family members may also represent an increased occurrence. In our study, 50 % of the persons with AS had difficulties in recognition of faces. This is in line with the study by Barton et al. [10], which showed that 2/3 of the persons with AS had heterogeneity in the perceptual or imagery processing of faces. Thus, these two lines of evidence support the assumption that individuals with AS have face recognition difficulties. Face recognition is one of the most complex visual tasks performed by the human brain [10]. According to the literature, persons with autism spectrum disorders process faces as if they were objects and appear to rely more on identifying individual pieces of the face rather than the overall configuration [27-29]. This is significant data, because recognition of faces is an important part of interpersonal interaction and successful functioning within a social group. These findings and the result of our study makes it attractive to speculate that the deficits in face perception may contribute to the impaired social communication in persons with AS. Therefore, including face recognition difficulties in the clinical criteria for AS may also be warranted, as it might represent a core dysfunction of AS. This is in line with the study by Barton et al. [10], which concludes the same point, that heterogeneity may be important to take into account in genetic investigations.

Aberrant sensory perception was a major finding in our study, especially in the domains of sound and touch. This is in agreement with the findings made by Dunn et al. [13,14] and Myles at al. [12], as it seems that the auditory and touch processing are the two most important aberrant sensory domains. The nature of aberrant auditory sensory processing in AS and autism is still open. According to Baranek [30], altered hyperreactions in auditory processing occur in autism in up to 100% of the cases. The auditory sensory processing was studied using mitch-match-negativity recording technique by Jansson-Verkasalo et al. [15] and clinically using the Sensory Profile by Dunn et al. [13,14]. The former study indicated that auditory sensory processing was deficient in children with AS. Dunn et al. [13,14] showed that children with AS had difficulties in auditory processing, but that these had more difficulty with auditory attention than children with autism. On the other hand, Čeponienė et al. [31], demonstrated auditory orienting deficits in autism, which could not be explained by sensory deficits and concluded that the orienting deficit in autism might be speech-sound specific. Auditory processing impairments in AS and autism deserve special attention, as they may constitute a part of the pathogenesis of the behavioral problems. On a general level, our observations of a change in sensory integration in the AS group are well in line with the results of corresponding studies of autism spectrum disorders [12-14,20,30,31].

Insomnia predisposes to emotional distress, daytime fatigue and a loss of productivity [18]. A careful assessment of sleep quality should therefore be integrated in the diagnostic interview for AS, as, according to our study, insomnia is a frequent finding in this group. The studies by Paavonen et al. [17] and Tani et al. [18] demonstrate an increased prevalence of disorders in initiating and maintaining sleep in both children and adults with AS, suggesting that sleep difficulties inherent to AS are preserved from childhood into adulthood. It is not known why persons with AS have insomnia or sleeping problems, but the reports by Godbout et al. [32,33] suggest that AS subjects may present the same difficulties in initiating and maintaining sleep as those previously described in autism. It has also been hypothesized that individuals with AS have a defective sleep control system which may be associated with the clinical picture of AS [33]. From a clinical point of view, sleep disturbances are typical among people with AS and should be taken into account when a person is referred for a diagnosis, especially as the problem can be alleviated by melatonin [17].

Our study recognized aberrant eating habits in 60 per cent of individuals with AS, which is much higher than the figure of approximately one per cent obtained by Sobanski et al. [21]. Eating problems [21,34,35] and also anorexia nervosa have been reported to be over-represented in AS [20,34-36]. As, there according to Gillberg et al. [19], is a 12 per cent co-morbidity of anorexia nervosa and AS and there frequently is a picky eating behavior it could be speculated that this is due to hypersensitivity, as low thresholds for taste in AS was shown by Dunn et al. [13,14]. This could be part of the explanation for a higher incidence rate of eating disorders among persons with AS.

## Conclusion

Our results speak in favour of the fact that aberrant sensory processing is an extensive phenomenon in AS, as these individuals frequently are hypersensitive to noise, bright lights, strong smells and touch. Our results are preliminary and need to be substantiated also using objective assessment methods before they can be seen as definite. Provided that the present findings can be substantiated in this way it might be possible to use the aberrant sensory processing also as a diagnostic criteria. It seems obvious that such aberrant sensory processing is a key feature of autism spectrum disorders, and our results will therefore probably not help in the differentiation between autism and AS.

## Authors' contributions

TN-vW was the main principal investigator collecting the data and the series. She was also principally responsible for preparing the manuscript. Together with author LvW and TY-O, she conceived the idea of this study

EJP participated in the design of the study and performed the statistical analysis

TY-0 was co-conceiver of the idea of this study and also in writing the manuscript

SS together with TK carried out all the ADI-R and/or ADOS investigations

TK together with SS carried out all the ADI-R and/or ADOS investigations

IJ participated in designing the study and in the writing of the manuscript

LvW helped to collect the data and the series. LvW also participated in designing the study and in the writing of the manuscript

## Note

**APPENDIX 1**. **The complementary questions regarding symptoms and signs not included in the ICD-10 and/or DSM-IV**.

*Asperger syndrome related additional features questionnaire (ASFQ)*

The following domains were included

*1. Presence of face recognition difficulties*

Do you have difficulty recognizing familiar persons from their faces?

Standardized clarifying questions were used, such as:

- If the clothing, color of the hair of an individual familiar to you has changed or you meet unexpectedly in an unfamiliar place, would you be able to recognize him/her?

- Do you generally recognize people on the basis of their style of walking, clothing, voice-/ speech rather than by their face?

*2. Presence of aberrant sensibilities*

The aim was to find out whether the individual considered him/herself and-/or was considered to represent hyper sensibility in the following modes of sensation:

- touch (light or strong)

- pain

- light

- external temperature

- sound

- smell

Fixed sets of clarifying questions were used.

*3. Sleeping/diurnal rhythm disturbances*:

The clarifying questions dealt with: getting to sleep, awakenings during the night, amount of sleep, abnormality of diurnal rhythm, use of sleeping pills, restless legs, impact of possible difficulties related to diurnal rhythm of every day life

*4. Aberrant eating habits*

The fixed clarifying questions comprised the following items:

- unusual-/odd food preferences

- unusual routines regarding arranging food on the plate and eating

- presence of aversion due to the smell of the food

- ongoing or past anorectic behavior

*5. Behavior*

The clarifying questions pinpointed possible presence of selection difficulties in ordinary every day situations (ice-cream a or b).

It was also established whether making up one's mind in such situation lead to avoidance of such situations. It was also made clear whether the person in such situations used clearly excessive time for making the decisions or whether these caused anxiety?

Another set of questions aimed at clarifying whether the individual him/herself recognized traits of hyperactivity and/or attention difficulties or whether other people had made him/her aware of such traits.

*6. External appearance*

Possible presence of unusual physical features with respect to stature, flexibility of joints and minor stigmata were recorded.

In both genders the questions aimed at finding out whether the individual frequently was wrongly perceived with respect to chronological age. Also the interviewer's perception of a possible youngish appearance was recorded.

In males questions also were focused on the age for starting regular shaving and the maximum interval between shavings which was compatible with socially acceptable tidiness of the face. Answers reporting such shaving less frequently than twice a week were considered being out of average range for adult Finnish men.

## Pre-publication history

The pre-publication history for this paper can be accessed here:


